# Distribution and clinical impact of apolipoprotein E4 in subjective memory impairment and early mild cognitive impairment

**DOI:** 10.1038/s41598-020-69603-w

**Published:** 2020-08-07

**Authors:** Hanna Cho, Young-Eun Kim, Wonjeong Chae, Ko Woon Kim, Jong-Won Kim, Hee Jin Kim, Duk L. Na, Chang-Seok Ki, Sang Won Seo

**Affiliations:** 1 Departments of Neurology, Samsung Medical Center, Sungkyunkwan University School of Medicine, 81 Irwon-ro, Gangnam-gu, Seoul, 135-710 Korea; 2 Department of Neurology, Gangnam Severance Hospital, Yonsei University College of Medicine, Seoul, Korea; 3 Laboratory Medicine and Genetics, Samsung Medical Center, Sungkyunkwan University School of Medicine, Seoul, Korea; 4 Department of Laboratory Medicine, Hanyang University College of Medicine, Seoul, Korea; 5 Department of Public Health, College of Medicine, Yonsei University, Seoul, Korea; 6 Department of Neurology, School of Medicine, Jeonbuk National University Hospital, Jeonju, Korea; 7 Neuroscience Center, Samsung Medical Center, Seoul, Korea; 8 Department of Clinical Research Design and Evaluation, SAIHST, Sungkyunkwan University, Seoul, Korea; 9 GC Genome, 107, Ihyeon-ro 30beon-gil, Giheung-gu, Yongin, Gyeonggi-do Korea

**Keywords:** Alzheimer's disease, Risk factors

## Abstract

The apolipoprotein E (APOE) e4 allele is the most common genetic variant associated with Alzheimer’s disease (AD). We sought to investigate the distribution of APOE genotypes across the full clinical AD spectrum including AD, late-stage amnestic mild cognitive impairment (L-aMCI), early-stage aMCI (E-aMCI), subjective memory impairment (SMI), and controls. We prospectively recruited 713 AD patients, 735 aMCI patients, 575 SMI patients, and 8,260 individuals as controls. The frequency of the APOE e4 allele revealed an ordered fashion in the AD (30.8%), L-aMCI (24.0%), E-aMCI (15.1%), SMI (11.7%), and control (9.1%) groups. APOE e3/e4 and e4/e4 genotype frequencies also appeared in an ordered fashion in the AD group (39.1% of e3/e4 and 10.9% of e4/e4), as well as the L-aMCI (28.3% and 9.4%), E-aMCI (22.3% and 3.7%), SMI (18.3% and 1.9%), and control (15.1% and 0.8%) groups. In the comparisons of APOE e3/e3 vs. e3/e4 genotypes, all patient groups had a higher frequency of APOE e3/e4 relative to the control group. Relative to the SMI and E-aMCI groups, the AD and L-aMCI groups had higher frequency of the APOE e3/e4 genotype, and the AD group had a higher frequency relative to the L-aMCI group. However, there was no significant difference between the E-aMCI and SMI groups. In our longitudinal data, APOE e4 carrier showed a steeper incline slope in a clinical dementia rating sum of boxes (CDR-SB) score than APOE e4 non-carrier in SMI (B = 0.0066, p = 0.0104), E-aMCI (B = 0.0313, p < 0.0001), and L-aMCI (B = 0.0178, p = 0.0007). APOE e4 carrier showed a steeper decline slope in the CDR-SB than APOE e4 non-carrier in AD (B = − 0.0309, p = 0.0003). These findings suggest that E-aMCI and SMI are associated with a similarly increased frequency of the APOE e4 allele compared to controls, suggesting a greater genetic risk for AD and the importance of monitoring the allele more closely.

## Introduction

The apolipoprotein E (APOE) e4 allele is the most common genetic variant associated with Alzheimer’s disease (AD), with the presence of an allele increasing the risk of developing AD^1–4^. Several studies have reported that the prevalence of APOE e4 is higher among patients with mild cognitive impairment (MCI) compared to the general population, at 36.5%^5^ and 27.9%^6^ in Western countries, and 21.4%^7^ in Asia.

Although the most commonly accepted prodromal AD stage is amnestic MCI (aMCI), recent studies (ADNI GO, ADNI 2) have described two forms based on severity: early aMCI (E-aMCI) and late aMCI (L-aMCI). L-aMCI refers to the original definition (with test performance of 1.5 SD below the normative mean), while E-aMCI is defined as between 1.0 and 1.5 SD below the normative mean on a memory test^8^. Furthermore, to define an even earlier point in time for disease detection, subjective memory impairment (SMI) is sometimes considered to be the first clinical indicator of AD even prior to aMCI. SMI is defined by subjective reports of memory decline with no evidence of cognitive impairment on formal testing^9^. Epidemiologic studies have reported more rapid progression in cognitive decline and the development of AD in E-aMCI^6^ and SMI patients^10–13^. Some studies have investigated the frequency of the APOE e4 allele in E-MCI in Western countries (NC:E-MCI = 24.4%:40.7%)^14^^,^ (NC:E-MCI = 18.9%:17.5%)^6^ and SMI (NC:SMI = 21%:29%)^15^^,^ (NC:SMI = 15%:22%)^5^^,^ (NC:SMI = 22.2%:26.8%)^16^^,^ as well as China (NC:SMI = 7.3%:14.7%)^7^. However, the results have been inconsistent, which could be due to the relatively small and varying sample sizes.

A greater understanding of the preclinical stages of AD is critical to determine the future development of AD. The aim of this study was to evaluate the distribution of the APOE e4 genotype in AD, L-aMCI, E-aMCI, SMI, and control groups in a relatively large cohort of patients across the full clinical AD spectrum, and to compare the frequency of the genotype between the different diagnostic categories. Furthermore, we determined whether APOE e4 genotype might affect clinical progression measured by a clinical dementia rating sum of boxes (CDR-SB) score in AD, L-aMCI, E-aMCI, and SMI.

## Results

### Demographic characteristics

The demographic characteristics of the study participants are listed in Table 1. The age at the initial visit recorded at the memory clinic was higher in the AD, L-aMCI, E-aMCI, and SMI groups compared to the control group. The gender distribution was distinct between the control and AD, L-aMCI, E-aMCI, and SMI groups. Mean MMSE scores were also lower in the AD group than in the other groups.

**Table 1:** Demographic characteristics.

	AD	L-aMCI	E-aMCI	SMI	Controls
No. of subjects (n)	713	434	215	575	8,260
Age	71.6 ± 10.1*^†^	71.7 ± 8.4*^†^	69.3 ± 8.1*	65.0 ± 9.3*	52.2 ± 10.6
Gender (f, %)	464 (65.1)*	253 (58.3)*^†^	140 (65.1)*	414 (72.0)*	1979 (24.0)
Education	9.2 ± 5.5	11.1 ± 4.6	9.8 ± 5.9	11.3 ± 5.1	N/A
MMSE	18.3 ± 5.8^†§¶^	25.6 ± 2.9^†^	26.1 ± 3.6	28.3 ± 2.2	N/A

*p < 0.05, vs. control group; ^†^*p* < 0.05, vs. SMI group; ^§^*p* < 0.05, vs. early aMCI group; ^¶^*p* < 0.05, vs. late aMCI group.

*AD* Alzheimer’s disease, *E-aMCI* early-stage amnestic mild cognitive impairment, *L-aMCI* late-aMCI, *N/A* not applicable, *SMI* subjective memory impairment.

### APOE genotypes and alleles

The distribution of the APOE allele differed between the diagnostic groups (Table 2). APOE e4 allele frequency had an ordered fashion in the AD, L-aMCI, E-aMCI, SMI, and control groups (30.8%, 24.0%, 15.1%, 11.7% and 9.1%, respectively).

**Table 2:** Distribution of APOE genotype and alleles by diagnostic group.

	AD	L-aMCI	E-aMCI	SMI	Controls
No. of subjects (n)	713	434	215	575	8,260
**Genotype (%)**					
2/2	0 (0.0)	1 (0.2)	0 (0.0)	5 (0.9)	25 (0.3)
3/2	34 (4.8)	24 (5.5)	16 (7.4)	66 (11.5)	891 (10.8)
3/3	318 (44.6)	242 (55.8)	142 (66.0)	380 (66.1)	5,909 (71.5)
2/4	4 (0.6)	3 (0.7)	1 (0.5)	8 (1.4)	107 (1.3)
3/4	279 (39.1)	123 (28.3)	48 (22.3)	105 (18.3)	1,261 (15.3)
4/4	78 (10.9)	41 (9.4)	8 (3.7)	11 (1.9)	66 (0.8)
**Alleles (%)**					
2	38 (2.7)	29 (3.3)	17 (4.0)	84 (7.3)	1,048 (6.3)
3	949 (66.5)	631 (72.7)	348 (80.9)	931 (81.0)	13,972 (84.6)
4	439 (30.8)	208 (24.0)	65 (15.1)	135 (11.7)	1,500 (9.1)

*APOE* apolipoprotein E, *AD* Alzheimer’s disease, *E-aMCI* early-stage amnestic mild cognitive impairment, *L-aMCI* late-aMCI, *SMI* subjective memory impairment.

APOE e3/e3 vs. e3/e4 genotyping was statistically significant between the diagnostic groups (Table 3). Relative to the control group, all patient groups had a higher frequency of the APOE e3/e4 allele. Relative to the SMI and E-aMCI groups, the AD and L-aMCI groups had a higher frequency of the APOE e3/e4 genotype, while the AD group had a higher frequency relative to the L-aMCI group. However, no statistical significance was observed between the E-aMCI and SMI groups.

**Table 3:** Statistical significance of APOE genotype frequency by diagnostic group (p values).

	AD	L-aMCI	E-aMCI	SMI	Controls
**E3/E3 vs. E3/E4**					
AD		< 0.05	< 0.05	< 0.05	< 0.05
L-MCI			< 0.05	< 0.05	< 0.05
E-MCI				0.313	< 0.05
SMI					< 0.05
**E3/E3 vs. E4/E4**					
AD		0.078	< 0.05	< 0.05	< 0.05
L-MCI			< 0.05	< 0.05	< 0.05
E-MCI				0.154	< 0.05
SMI					< 0.05
**E4 carrier vs non-E4 carrier**					
AD		< 0.05	< 0.05	< 0.05	< 0.05
L-MCI			< 0.05	< 0.05	< 0.05
E-MCI				0.140	< 0.05
SMI					< 0.05

The FDR method was used for multiple comparisons between the groups.

*APOE* apolipoprotein E, *AD* Alzheimer’s disease, *E-aMCI* early-stage amnestic mild cognitive impairment, *L-aMCI* late-aMCI, *SMI* subjective memory impairment.

Comparison of the APOE e3/e3 vs. e4/e4 genotypes or APOE e4 carrier vs. APOE e4 non-carriers showed a similar pattern to comparisons of the APOE e3/e3 vs. e3/e4 genotypes (Table 3).

### Longitudinal cognitive change according to APOE e4 genotypes

**Figure 1:**
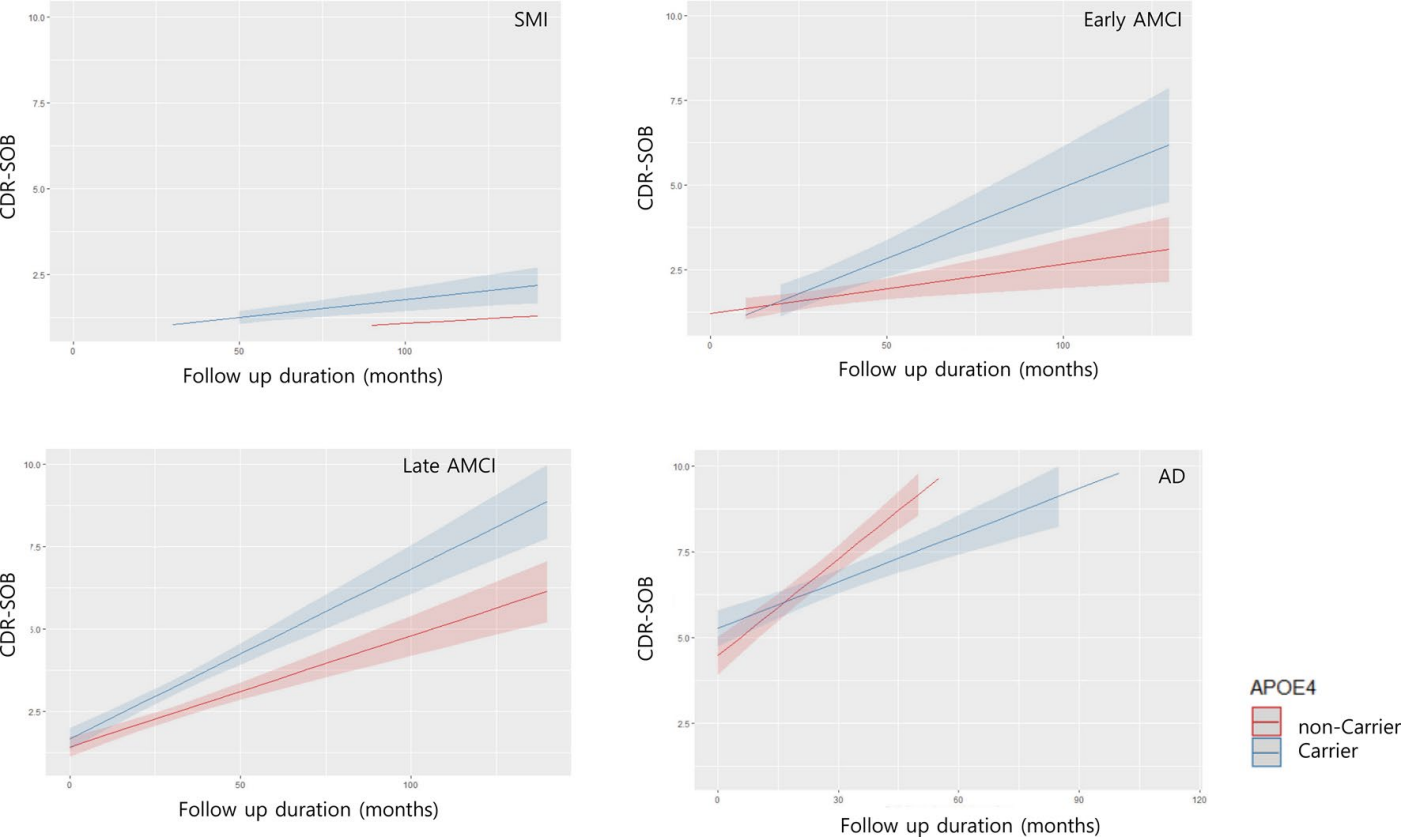
Cognitive changes in the follow-up duration according to APOE e4 genotypes. APOE e4 carrier showed a steeper incline slope in CDR-SB score than APOE e4 non-carriers in SMI, E-aMCI, and L-aMCI. However, in AD, APOE e4 non-carrier showed a steeper inline slope in the CDR-SB than APOE e4 carriers. *APOE* apolipoprotein E, *CDR-SB* clinical dementia rating sum of boxes, *AD* Alzheimer’s disease, *E-aMCI* early-stage amnestic mild cognitive impairment, *L-aMCI* late-aMCI, *SMI* subjective memory impairment.

Supplementary Table S2 summarizes the characteristics of the subjects included in the longitudinal study. APOE e4 carrier showed a steeper incline slope in a clinical dementia rating sum of boxes (CDR-SB) score than APOE e4 non-carriers in SMI (B = 0.0066, p = 0.0104), E-aMCI (B = 0.0313, p < 0.0001), and L-aMCI (B = 0.0178, p = 0.0007) (Figure). APOE e4 carrier showed a steeper decline slope in the CDR-SB than APOE e4 non-carriers in AD (B = -0.0309, p = 0.0003) (Fig. 1).

## Methods

### Subjects

We prospectively recruited 713 AD patients, 735 aMCI patients, and 575 SMI patients, with all diagnosed at Samsung Medical Center (Seoul, Republic of Korea) from August 2006 until June 2012. Eligible patients were required to meet the criteria for probable AD as described by NINCDS-ADRDA (National Institute of Neurological and Communicative Disorders and Stroke and the Alzheimer’s Disease and Related Disorders Association)^17^. All aMCI cases met the criteria described by Petersen et al.^18^, including: (1) subjective memory complaint by patients or caregivers; (2) normal general cognitive function as defined by scores on the Korean version of the Mini-Mental State Examination (MMSE) ≥  − 1.0 standard deviation (SD) of the norms for age- and education-matched normal participants; (3) normal activities of daily living (ADL), as judged both clinically and on the ADL scale described later; (4) objective memory decline below the sixteenth percentile (− 1.0 SD) on either verbal or visual memory tests; and (5) without dementia. The aMCI subjects were classified in accordance with the baseline results of their memory test. Abnormal memory function was classified when recall scores were delayed on either the SVLT or RCFT at lower than − 1.0 SD relative to age-, gender-, and education- matched norms. Patients with scores that were between − 1.5 and − 1.0 SD of age-, gender-, and education-matched norms received an E-aMCI classification, while scores lower than − 1.5 SD resulted in an L-aMCI classification. From the 735 patients with aMCI, we excluded 86 subjects with incomplete data from the neuropsychological tests. Finally, 649 patients with aMCI were further classified E-aMCI (215) and L-aMCI (434) groups. SMI was determined if the memory clinic received a referral for subjects for the reason of memory impairment and by the standard question: “Do you feel like your memory is becoming worse?” with the answer “Yes, this worries me”. Subjects were excluded if they selected either of the other options: “no” or “yes, but this does not worry me”. To support the validity of memory impairment, only those subjects whose memory worsening was confirmed by others, including their spouses or close relatives (often accompanying the patient to the memory clinic). SMI was defined by subjective memory complaint from patients or caregivers, as well as normal cognitive function as defined by objective neuropsychological test results, normal ADL, and without mild cognitive impairment. Cognitive function was considered normal when all cognitive tests were higher than − 1.0 SD of the age-, gender-, and education- matched norms.

After obtaining informed consent, all patients were required to undertake comprehensive interviews, a neurological exam, and a series of neuropsychological tests [Seoul Neuropsychological Screening Battery (SNSB)]^19–21^, brain MRI scans, and APOE e4 genotyping. Patients were excluded from the study if they presented with other structural lesions detected on brain MRI, including territorial infarction, brain tumor, intracranial hemorrhage, hydrocephalus or severe white matter hyperintensities (WMH). The possibility of alternative causes of cognitive deficits was eliminated by laboratory testing for complete blood count, blood chemistry, folate, vitamin B12, syphilis serology and thyroid function tests. The study protocol was approved by Samsung Medical Center’s Institutional Review Board. All methods in this study were performed in accordance with applicable guidelines.

### APOE genotyping

The Wizard Genomic DNA Purification kit was used to extract genomic DNA from peripheral blood leukocytes, following the manufacturer’s instructions (Promega, Madison, WI, USA). Within the *APOE* gene, the single nucleotide polymorphisms (rs429358 in codon 112 and rs7412 in codon 158) were genotyped with a TaqManSNP Genotyping Assay (Applied Biosystems, Foster City, CA, USA) using a 7500 Fast Real-Time PCR System (Applied Biosystems) according to the manufacturer’s instructions.

In order to compare the *APOE* genotype and allele frequencies of AD, MCI, and SMI groups with a control population, an extensive dataset including 8,260 individuals who underwent routine health exams between 1995 and 2002 at Samsung Medical Center’s Health Promotion Center was used.

### Neuropsychological testing

The patients were required to undergo neuropsychological testing using the Seoul Neuropsychological Screening Battery (SNSB)^19–21^. The battery is comprised of quantitative tests, including the Korean version of the Boston Naming Test (K-BNT)^22^^,^ digit span (forward and backward), Rey-Osterrieth Complex Figure Test (copying, immediate, 20-min delayed recall, and recognition), phonemic and semantic Controlled Oral Word Association Test (COWAT), Seoul Verbal Learning Test (SVLT; three learning-free recall trials of 12 words, a 20-min delayed recall trial for these 12 items, and a recognition test), and a Stroop Test (word/color reading of listed items over a 2-min period).

### Clinical follow-up

We collected CDR-SB scores from 455 patients (82 SMI, 61 E-aMCI, 169 L-aMCI, and 143 AD) who were followed up more than three times to obtain CDR-SB scores at the Samsung Medical Center. Their clinical follow-up was performed until 2015. The study subjects were examined for 2.4 ± 2.2 years from baseline. Although the follow-up times and durations varied among participants, their mean duration was 29.2 months while the follow-up tests were conducted 4.0 times on average.

### Statistical analysis

Chi-square test was applied to assess the *APOE* genotype and allele frequencies among the AD, L-aMCI, E-aMCI, SMI and control groups. Statistical significance was defined at *p* < 0.05, corrected for the false discovery rate (FDR).

Longitudinal statistical analyses were conducted with linear mixed effect model and adjusted for age, sex, education years, follow-up duration and presence of an APOE e4 (PROC MIXED; SAS version 9.4, SAS Institute).

## Discussion

Few studies have investigated the frequency of APOE e4 alleles in the continuum of NC and AD in Asia^7^^,^ although some similar studies in Western countries have been reported^5,6^. In the present study, an ordered trend in the frequency of the APOE e4 allele was observed: AD (30.8%), L-aMCI (24.0%), E-aMCI (15.1%), SMI (11.7%), and control (9.1%) groups, suggesting that there is an increasing trend in the frequency of APOE e4 alleles in the AD spectrum. Furthermore, APOE e4 carrier showed more rapid cognitive decline than APOE e4 non-carrier from SMI to L-aMCI while in AD, APOE e4 non-carrier showed more rapid cognitive decline than APOE e4 carrier. Therefore, our findings replicated that APOE e4 genotype is a prognostic factor of AD as well as important risk factor.

We investigated the distribution of APOE genotypes in AD, L-aMCI, E-aMCI, SMI, and control groups in Korea. Across all groups, we observed increasing frequency of the APOE e4 allele in an ordered fashion, with the highest risk in AD (30.8%) followed by L-aMCI (24.0%), E-aMCI (15.1%), SMI (11.7%), and control (9.1%) groups (Table 2). This finding shows that frequency of APOE e4 allele increases as impairment increases. The incidence of the APOE ε4 allele in patients with AD was consistent with the findings of previous studies in Korea which reported 44.3%^23^^,^ 22.3%^24^^,^ and 41.6%^25^. The frequency of the APOE e4 with NC was also comparable to that found in normal Korean subjects (9.1%) in another study^24^. Previous studies have demonstrated consistent results with the frequency of the APOE e4 allele in L-aMCI being significantly higher compared with the general population: 36.5%^5^ and 27.9%^6^ in Western countries, and 21.4% in China^7^. In addition, several reports have demonstrated that the APOE e4 genotype is associated with a more rapid progression from L-aMCI to AD^26–28^, implying that the presence of APOE e4 is a genetic risk factor for the future development of AD.

We have found that APOE e4 frequency in ethnic Koreans is lower than that reported in Western studies for controls as well as AD and MCI patients. Previous studies have consistently shown that patients of Asian ethnicity have lower APOE e4 frequency than their Western counterparts with AD and MCI^29,30^ and in the general population^29,31^. In a recent meta-analysis^32^^,^ the frequency of APOE e4 carriers was 68.9% vs 52.1% in AD, 52.5% vs 33.3% in MCI, and 35.3% vs 22.5% in control subjects (northern Europe vs Asia)^32^. They demonstrated that APOE e4 prevalence in Asia was statistically lower than in northern Europe and North America. However, because the number of Asians in the study was relatively small (around 100 per group), it was necessary to assess the frequency of APOE e4 in an additional larger cohort. Our results suggest that the frequency of APOE e4 can be dependent on racial and regional differences, which should be considered in clinical research and during the selection of patients for AD treatment.

We observed that the frequency of the APOE e3/e4 genotype is higher in E-aMCI and SMI as well as AD and L-aMCI, relative to the controls (Table 3). There have been previous reports on the frequency of APOE e4 compared to normal controls in E-MCI (NC:E-MCI = 24.4%:40.7%)^14^^,^ (NC:E-MCI = 18.9%:17.5%)^6^ and SMI (NC:SMI = 21%:29%)^15^^,^ (NC:SMI = 15%:22%)^5^^,^ (NC:SMI = 22.2%:26.8%)^16^ in Western countries, and in China (NC:SMI = 7.3%:14.7%)^7^. However, the results have been inconsistent, possibly due to the relatively small and varying sample sizes. In the present study, we confirmed that the frequency of the APOE e3/e4 genotype in the E-aMCI and SMI groups was significantly higher than in normal controls, indicating that people with an early stage of objective memory impairment or with subjective memory complaints may be at increased risk for AD.

AD prevalence increases exponentially as age increases, so dementia factors have a high age distribution, while the controls belong to a lower age group that is not yet at risk of dementia, so it may seem that the control groups have lower APOE e4 prevalence. As outlined in previous studies, various methods have been used for the application of age distribution, including Bayesian probabilities and Gompertz Law^33–35^. Therefore, for the association between APOE genotype and disease stage, taking into account the confounding effect of age distribution within the disease stage groups in our study, we stratified the data by age group and applied the Cochran–Mantel–Haenszel (CMH) test (Supplemental Table S1). Following this (Supplemental Table S1), we found almost consistent outcomes with the previous results without consideration for age (Table 3) other than the comparison of the APOE e3/e3 and e3/e4 genotypes between E-aMCI and controls.

Interestingly, we found no significant difference in the frequency of the APOE e3/e3 vs. e3/e4 genotypes between the E-aMCI and SMI groups (Table 3). To our knowledge, only one study has reported on the comparison of the APOE e4 frequency between patients with early MCI (17.5%) and SMI (19.8%)^6^. This study found that the two groups did not differ in the frequency of the APOE e4, which is consistent with our results. However, this previous study had some limitations, as they focused on an AD biomarker using neuroimaging and neuropsychological tests, rather than a genetic test, and as they partially enrolled participants for the study of APOE e4, the sample size was smaller than for our study. There were no significant differences in the frequency of the APOE e4 allele even in the early MCI (17.5%) or SMI (19.8%) groups, compared with the control group (18.9%).

Exactly why there is no significant difference in the APOE e4 genotype between E-aMCI and SMI remains unclear. A previous study suggests that clinically diagnosed aMCI patients might be pathologically heterogeneous^36^^,^ and it is also known that the presence of anxiety or depression may negatively impact memory performance^37^. It is possible that E-aMCI group included patients other than the early stage of AD, and therefore the subjects with E-aMCI performing between 1.0 SD and 1.5 SD below the norm on memory tests have no additional effect on the frequency of the APOE e4 genotype in our data. Furthermore, we cannot exclude the possibility that a categorical definition of E-aMCI as a minimal impairment is unsuitable to detect individuals at the earliest AD stages. Based on our results, it remains unclear whether SMI and E-aMCI can be clearly separated, and thus these two groups could be considered to be a continuum in the middle stage between normal cognition and MCI.

Nevertheless, our finding may have important clinical implications. The results of the frequency of APOE e4 is comparable among patients with E-aMCI and SMI suggesting that subjective complaints, even in the absence of objective impairment, may be a precise indicator of early disease-related changes as much as a single time point measurement of minimal memory impairment. Our suggestion is supported by a previous study showing that SMI and E-aMCI are associated with a similarly increased risk of AD dementia^6^. Previous studies have also suggested that subjective complaints are associated with the future development of dementia^10–13,38^. Overall, our data strengthens the importance of the subjective experience of memory impairment in dementia prediction.

Another major finding was that APOE e4 genotyping is one of the most predictive factors of cognitive decline from SMI to L-aMCI while once patients progress to clinically manifest AD, the effects of APOE e4 genotyping disappeared. Our findings were consistent with previous studies showing that APOE e4 genotype is the most important genetic risk factor for developing AD^39^. However, after patients progress to clinically manifest dementia, the effects of APOE e4 genotype are less clear^40^. Furthermore, a previous study showed that APOE e4 non-carriers with AD dementia have more amyloid burdens than carriers^41^ although many studies have shown that APOE e4 carriers more amyloid burdens than non-carriers in participants with normal cognition and MCI^42^. Our findings of the APOE e4 non-carriers on more rapid cognitive decline in AD might be explained by APOE e4 carriers having less brain reserve. That is, since pathology in APOE e4 non-carriers is more severe than in APOE e4 carriers after AD has developed, there is much less substrate remaining to function as a reserve^43,44^. Therefore, the insufficient reserve in APOE e4 non-carriers contributes towards accelerated deterioration of AD^45–47^.

We note some limitations to our findings. Our patients were selected from a single center, which may limit the generalizability of the findings, and we used a cross-sectional study design. In addition, the study lacks CSF, blood biomarker and imaging data. Further longitudinal and multi-center prospective studies are needed to better understand the actual risk of developing AD and the rate of disease progression in the AD spectrum for participants with or without APOE e4.

In conclusion, we found that E-aMCI and SMI are associated with a similarly increased frequency of the APOE e4 allele, suggesting a greater genetic risk for AD and the importance of monitoring the allele more closely.

## Supplementary information

Supplementary file1

## Data Availability

The data that support the findings of this study are available on request from the corresponding author. The data are not publicly available due to privacy restriction.
