# Supplementary material for: Distribution and clinical impact of apolipoprotein E4 in subjective memory impairment and early mild cognitive impairment
Source: Sci Rep. 2020 Aug 7;10:13365. doi: 10.1038/s41598-020-69603-w (PMC7414226; doi:10.1038/s41598-020-69603-w)
Supplement: Supplementary file 1 — Supplementary file1 [file 41598_2020_69603_MOESM1_ESM.docx]

**[Supplementary information]**

**Distribution and Clinical Impact of Apolipoprotein E4 in Subjective Memory Impairment and Early Mild Cognitive Impairment**

Hanna Cho^1, 2, a^, Young-Eun Kim^3, a^, Wonjeong Chae, ^4^ Ko Woon Kim, ^5^ Jong-Won Kim^3^,

Hee Jin Kim^1^, Duk L. Na^1,6,7^, Chang-Seok Ki^3**^, Sang Won Seo^1,6,7*^

Departments of ^1^Neurology and ^3^Laboratory Medicine & Genetics, Samsung Medical Center, Sungkyunkwan University School of Medicine, Seoul, Korea

^2^Department of Neurology, Gangnam Severance Hospital, Yonsei University College of Medicine, Seoul, Korea

^4^Department of Public Health, College of Medicine, Yonsei University, Seoul, Korea

^5^Department of Neurology, School of Medicine, Jeonbuk National University Hospital, Jeonju, Korea

^6^Neuroscience Center, Samsung Medical Center, Seoul, Korea

^7^Department of Clinical Research Design and Evaluation, SAIHST, Sungkyunkwan University, Seoul, Korea

^a^Two authors equally contributed to this study.

^*^Two corresponding authors equally contributed to this study.

**Correspondence to:**

^*^**Sang Won Seo, M.D., Ph.D.** Department of Neurology, Samsung Medical Center, Sungkyunkwan University School of Medicine, 81 Irwon-ro, Gangnam-gu, Seoul, 135-710, Korea. Tel: 82-2-3410-1397, fax: 82-2-3410-0052, e-mail: sangwonseo@empal.com

^**^**Chang-Seok Ki, M.D., Ph.D.** Department of Laboratory Medicine and Genetics, Samsung Medical Center, Sungkyunkwan University School of Medicine, 81 Irwon-ro, Gangnam-gu, Seoul, 135-710, Korea. Tel: 82-2-3410-2709, fax: 82-2-3410-2719, e-mail: changski@skku.edu

Supplemental Table 1. Statistical significance of APOE genotype frequency by diagnostic group applied Cochran-Mantel-Haenszel test (p values)

| **E3/E3 vs. E3/E4** | **AD** | **L-aMCI** | **E-aMCI** | **SMI** | **Controls** |
| --- | --- | --- | --- | --- | --- |
| AD |  | < 0.05 | < 0.05 | < 0.05 | < 0.05 |
| L-MCI |  |  | < 0.05 | < 0.05 | < 0.05 |
| E-MCI |  |  |  | 0.267 | 0.117 |
| SMI |  |  |  |  | < 0.05 |
| **E3/E3 vs. E4/E4** | **AD** | **L-aMCI** | **E-aMCI** | **SMI** | **Controls** |
| AD |  | 0.078 | < 0.05 | < 0.05 | <0.05 |
| L-MCI |  |  | < 0.05 | < 0.05 | <0.05 |
| E-MCI |  |  |  | 0.282 | <0.05 |
| SMI |  |  |  |  | < 0.05 |
| **E4 carrier vs.  non-E4 carrier** | **AD** | **L-aMCI** | **E-aMCI** | **SMI** | **Controls** |
| AD |  | < 0.05 | < 0.05 | < 0.05 | < 0.05 |
| L-MCI |  |  | < 0.05 | < 0.05 | < 0.05 |
| E-MCI |  |  |  | 0.129 | < 0.05 |
| SMI |  |  |  |  | < 0.05 |

The FDR method was used for multiple comparisons between the groups.

Abbreviations: AD = Alzheimer’s disease; E-aMCI = early-stage amnestic mild cognitive impairment; L-aMCI = late-aMCI; SMI = subjective memory impairment

Supplemental Table 2. The characteristics of the subjects included in the longitudinal study

|  | **AD** | **L-aMCI** | **E-aMCI** | **SMI** |
| --- | --- | --- | --- | --- |
| No. of subjects (n) | 143 | 169 | 61 | 82 |
| Baseline age (year), median (IQR) | 74.0(9.4) | 73.0(8.0) | 71.0(7.9) | 68.4(6.9) |
| Gender (f, %) | 95(66.4) | 106(61.0) | 38(62.3) | 60(73.2) |
| Education (year), median (IQR) | 9.0(5.3) | 12.0(4.7) | 12.0(5.5) | 12.0(5.6) |
| Baseline CDR-SB, median (IQR) | 5.5(3.3) | 2.0(1.9) | 1.0(1.9) | 0.5(0.8) |
| APOE $\varepsilon$4 carries, no (%) | 73(51.1) | 75(44.4) | 15(24.6) | 26(31.7) |
| Number of visits, median (IQR) | 4.0(1.3) | 4.0(1.4) | 4.0(1.6) | 4.0(1.3) |
| Follow up month, median (IQR) | 23.2(22.7) | 23.6(24.0) | 23.5(27.3) | 34.3(34.5) |

Abbreviations: AD = Alzheimer’s disease; E-aMCI = early-stage amnestic mild cognitive impairment; L-aMCI = late-aMCI; SMI = subjective memory impairment; IQR = Inter-Quartile Range
